# Monitoring respiratory muscles effort during mechanical ventilation

**DOI:** 10.1097/MCC.0000000000001229

**Published:** 2024-11-14

**Authors:** Julien P. van Oosten, Evangelia Akoumianaki, Annemijn H. Jonkman

**Affiliations:** a Intensive Care Volwassenen, Erasmus Medical Center, Rotterdam, The Netherlands; b Adult Intensive Care Unit, University Hospital of Heraklion, Heraklion; c Medical School, University of Crete, Heraklion, Greece

**Keywords:** esophageal manometry, expiratory muscles, lung stress, occlusion pressure, respiratory muscles effort

## Abstract

**Purpose of review:**

To summarize basic physiological concepts of breathing effort and outline various methods for monitoring effort of inspiratory and expiratory muscles.

**Recent findings:**

Esophageal pressure (Pes) measurement is the reference standard for respiratory muscle effort quantification, but various noninvasive screening tools have been proposed. Expiratory occlusion pressures (P0.1 and Pocc) could inform about low and high effort and the resulting lung stress, with Pocc outperforming P0.1 in identifying high effort. The pressure muscle index during an inspiratory hold could unveil inspiratory muscle effort, however obtaining a reliable inspiratory plateau can be difficult. Surface electromyography has the potential for inspiratory effort estimation, yet this is technically challenging for real-time assessment. Expiratory muscle activation is common in the critically ill warranting their assessment, that is, via gastric pressure monitoring. Expiratory muscle activation also impacts inspiratory effort interpretation which could result in both under- and overestimation of the resulting lung stress. There is likely a future role for machine learning applications to automate breathing effort monitoring at the bedside.

**Summary:**

Different tools are available for monitoring the respiratory muscles’ effort during mechanical ventilation – from noninvasive screening tools to more invasive quantification methods. This could facilitate a lung and respiratory muscle-protective ventilation approach.

## INTRODUCTION

**FIGURE 1:**
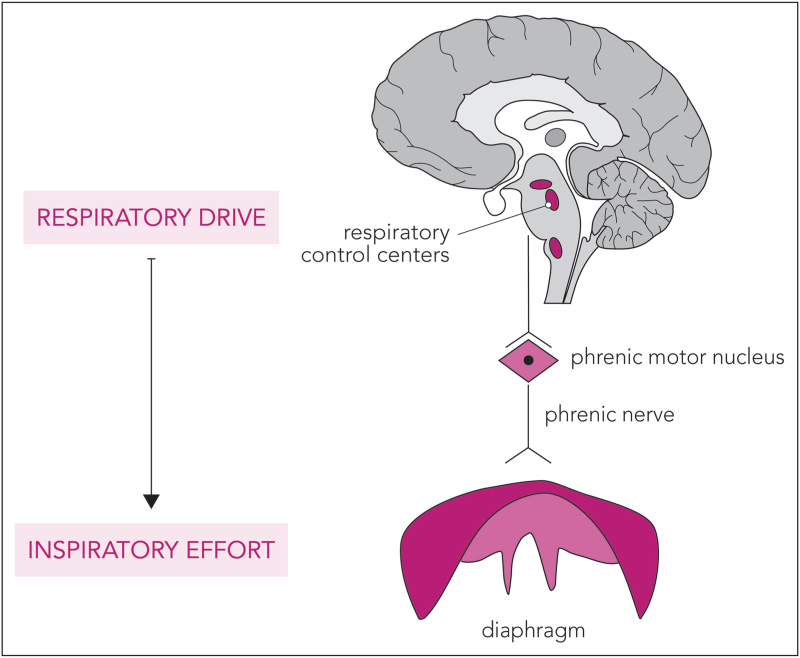
Respiratory drive and effort physiological pathway. Respiratory drive reflects the output of the respiratory centers in the brainstem. Via the phrenic nerve motor units this results in activation of the diaphragm and force generation.

Respiratory effort reflects the energy-consuming activity of the respiratory muscles that drive respiration. Its magnitude is primarily controlled by the intensity of the output of the respiratory centers in the brainstem, that is, respiratory drive [1,2] (Fig. 1). Drive and effort are distinct, yet closely associated physiological entities: when the phrenic nerve and diaphragm function are intact, high drive results in high effort. However, in the presence of respiratory muscle dysfunction, which is common in the critically ill [3], a high respiratory drive results in low inspiratory effort, contributing to dyspnea sensation [4]. 

**Box 1:**
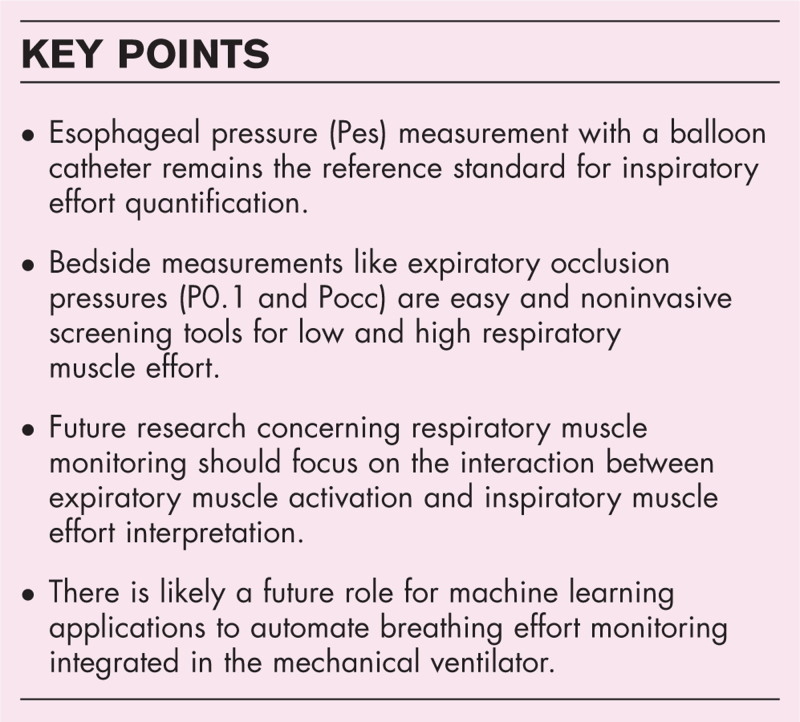
no caption available

Understanding the physiology of breathing effort is essential for individualizing ventilator settings to provide lung and respiratory muscle-protective ventilation [4,5]. Indeed, both too low and excessive respiratory drive and effort have been linked to diaphragm dysfunction [6,7], and excessive effort could result in high (regional) lung distending pressures (e.g., via pendelluft [8]) and potentially self-inflicted lung injury [4,5,9]. Convincing evidence regarding the impact of high effort on the diaphragm and lung injury is still lacking; however, this also highlights the need for reliable easy-to-use monitoring tools to assess such impact.

This review describes the basic physiological concepts of breathing effort and outlines various methods for monitoring respiratory muscle effort during mechanical ventilation, focusing on recent developments. We also address the role of the expiratory muscles in detail, since it is increasingly recognized that expiratory muscle activation is common in the critically ill [10] warranting their assessment, and also impacts inspiratory effort monitoring and interpretation [11^▪▪^,^12^].

## PHYSIOLOGY OF BREATHING EFFORT

The respiratory muscle pump must generate effort to overcome both the resistive pressure of the airways and the elastic pressures of the respiratory system to achieve alveolar ventilation. Esophageal pressure (Pes) measurement as a surrogate for pleural pressure allows to separate the respiratory system elastic pressure between that used to stretch the lungs [i.e., transpulmonary pressure (P_L_)] and that required to expand the chest wall [13^▪▪^].

In spontaneous breathing, inspiratory effort is primarily generated by the diaphragm. Accessory inspiratory muscles are recruited with exercise or disease and include the parasternal, external intercostal, scalene, and sternocleidomastoid muscles [14]. Activation of inspiratory muscles results in negative pleural and airway pressure with respect to the atmospheric pressure; this pressure gradient drives flow into the lower respiratory tract and thus generates lung inflation. Expiration is typically passive; however, in the presence of high load imposed on the respiratory system or low inspiratory muscle capacity, expiratory muscles are recruited, including the lateral abdominal wall muscles and the internal intercostal muscles [10]. Active expiration is a critical mechanism to increase tidal volume beyond the capability of inspiratory muscle effort alone [15].

During mechanical ventilation, the ventilator partially unloads the respiratory muscles depending on the amount of supportive pressure provided or completely unloads the respiratory muscles in a deeply sedated patient on mandatory ventilation. Tidal volume in assisted ventilation thus depends on ventilator assistance *and* patient effort, meaning that monitoring ventilator waveforms alone does not inform about the actual patient's effort and risks of high lung distending pressures.

## MONITORING INSPIRATORY EFFORT

Different techniques have been proposed to evaluate the force output of the respiratory muscles and are summarized in Table 1.

**Table 1:** Overview of monitoring tools for inspiratory and expiratory muscle effort

Monitor tool	Muscle group	(Non)invasiveness	Cut-off values	Advantages	Comments
Clinical signs: accessory or abdominal muscle use, respiratory rate, rapid shallow breathing index (RSBI)	Inspiratory muscles, expiratory muscles	Noninvasive	-	Easy to perform first screening of increased respiratory work.	Changes in respiratory rate poorly reflect changes in respiratory drive.
Expiratory occlusion: P0.1	Inspiratory effort	Noninvasive	Low effort: P0.1 <1.0 cmH_2_O. High effort: P0.1 >3.5–4.0 cmH_2_O.	Requires only a simple maneuver or automatically calculated on ventilators. P0.1 primarily reflects inspiratory drive. Good screening tool for low drive and effort; moderate screening tool for identifying high drive and effort [17].	Automated P0.1 measurements by ventilators could underestimate effort. Affected by expiratory muscle relaxation. Severe impairment of respiratory system mechanics or muscle strength will affect P0.1 validity as index of effort.
Expiratory occlusion: Pocc	Inspiratory effort	Noninvasive	Proposed normal/target values between −3 to −15 cmH_2_O, depending on the predicted ΔP_L_. If predicted ΔP_L_ is >20 cmH_2_O, lung stress is likely high. Formulas: Predicted Pmus = –3/4 Pocc Predicted ΔP_L_ = (Ppeak – PEEP) – 2/3 Pocc	Easy to perform bedside as screening tool for inspiratory effort. Can be used to estimate muscle effort and lung stress (ΔP_L_) [20,21^▪▪^].	Pocc demonstrated better performance for identifying high effort as compared to P0.1 [21^▪▪^]. Affected by expiratory muscle relaxation. Not available automatically in all ventilators/modes (i.e., volume-assist control, BiLevel).
Inspiratory occlusion: pressure muscle index (PMI)	Inspiratory effort	Noninvasive	Passive patient: PMI <0 cmH_2_O Higher PMI corresponds with higher effort, but cut-off values should be established. The absence of a stable plateau during the inspiratory hold indicates high effort.	Low PMI could identify over-assistance. Informs about driving pressure.	Criteria for a reliable plateau have been described [12], but a stable inspiratory hold cannot assure the absence of expiratory muscle activity.
(Surface) electromyography ((s)EMG)	Inspiratory effort	Noninvasive	Not available.	(Future) potential for continuous monitoring.	(s)EMG amplitudes do not inform about effort: a conversion factor is needed to relate electrical activity to force output. Technically challenging for real-time assessment of Pmus.
Esophageal and/or gastric manometry (Pes, Pmus, Pdi, Pga)	Inspiratory effort (Pes, Pdi, Pmus), expiratory effort (Pga)	Invasive	Proposed normal/target values: ΔPes 3–12 cmH_2_O ΔPdi 3–15 cmH_2_O Pmus 3–15 cmH_2_O ΔPga ≥1–2 cmH_2_O during expiration can be considered expiratory muscle use.	Reference standard for quantifying respiratory muscle effort. Pga monitoring allows better interpretation of inspiratory effort in the presence of expiratory muscle use and is necessary to assess the diaphragmatic effort.	Needs dedicated equipment and technical expertise. No validated method for gastric balloon calibration exists. Allows computation of e.g., work of breathing, pressure-time-product.
Automated analyses of flow-time and Paw-time curves	Inspiratory effort, expiratory effort	Noninvasive	Yet to be established.	Could allow continuous monitoring.	Future potential and topic of research.

### Esophageal manometry

Pes measurement remains the reference standard for monitoring inspiratory effort. The application of the technique has been extensively summarized recently [13^▪▪^]. Nowadays Pes can be easily visualized in real-time by connecting the esophageal balloon to the ventilator or bedside monitor. The drop in Pes during spontaneous efforts, that is, ΔPes, or Pes swing, reflects a decrease in pleural pressure as generated by inspiratory muscle contraction (diaphragm + accessory inspiratory muscles). It should be noted that the total respiratory muscle pressure (Pmus) during inspiration also includes the pressure that is generated to expand the chest wall. However, this requires knowledge of the chest wall elastance, which is impossible to accurately measure during active breathing. It is regarded that values measured during passive ventilation could be used, or that chest wall elastance can be roughly estimated as 4% of predicted vital capacity [16].

When Pes measurement is combined with gastric pressure (Pga) monitoring using a double-balloon nasogastric catheter, the transdiaphragmatic pressure (Pdi = Pga – Pes) can be obtained as a diaphragm-specific measure of effort.

More complex measurements of effort can be obtained from Pes, Pmus, and Pdi tracings by accounting for the duration of contraction or volume displacement, including: work-of-breathing (area under the tidal volume–pressure curve), pressure-time-product (pressure integrated over the duration of effort) or pressure-rate-product (pressure swing multiplied by respiratory rate). These methods are mostly limited to research applications.

### Noninvasive surrogates

#### Occlusion pressures

**FIGURE 2:**
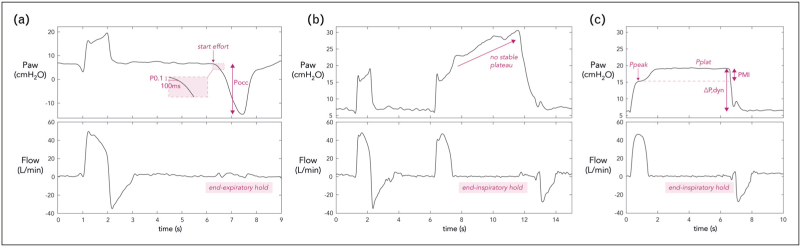
(a) End-expiratory hold maneuver and resulting occlusion pressure measurements in a patient on pressure-support ventilation. P0.1 reflects the drop in airway pressure (Paw) during the first 100 ms of an occluded inspiration, while the full breath occlusion pressure (Pocc) is the drop in Paw during a full occluded inspiratory effort. This example also shows the potential discrepancy between both methods and that Pocc better reflects high effort than P0.1: in this patient, P0.1 was 0.9 cmH_2_O, while Pocc was −21 cmH_2_O. (b) Inspiratory hold maneuver in a patient on pressure-support ventilation (same patient as in a). No stable plateau phase could be reached, seen as a continuous increase in airway pressure (Paw) during the inspiratory hold, making assessment of the pressure muscle index (PMI) impossible. This could in itself be a sign of high inspiratory effort – in line with this patient's Pocc assessment. (c) Theoretical illustration of how a reliable PMI measurement could have looked like in this patient in the absence of high effort. PMI is then measured as the difference between the stable plateau pressure (Pplat) minus the peak Paw (Ppeak). The dynamic airway driving pressure (ΔP,dyn) can then also be monitored as Pplat minus the positive end-expiratory pressure.

Two noninvasive measures for drive and effort that can be intermittently derived from the Paw-waveform are the P0.1 and the occlusion pressure (Pocc) (Fig. 2a). P0.1 is primarily a surrogate of the respiratory drive [17,18], measured as the airway pressure drop during the first 0.1 s of an occluded inspiration. Because of this short duration, there is no conscious response of the patient to this occlusion and susceptibility to the impairment of respiratory mechanics or neuromuscular dysfunction is limited, except in cases of severe derangement [18]. P0.1 correlates moderately with diaphragm electrical activity (healthy subjects, *R*^2^ = 0.51 for between-subject correlation) and reasonably well with inspiratory effort (patients, *R*^2^ = 0.67 for between-subject correlation of P0.1 and Pes-time-product) [17]. It was found especially useful for identifying extremes of drive and effort, with P0.1 <1.0 cmH_2_O as a good screening tool for low respiratory drive and P0.1 >3.5–4.0 cmH_2_O reflecting high drive and effort moderately well [17]. P0.1 can be obtained directly on the ventilator, but different ventilators use different algorithms, for example, the pressure drop during a 0.1 s occlusion (manual selection of P0.1 maneuver), or as automatically extrapolated from the slope of the pressure drop during the triggering phase. Therefore, the value could sometimes be underestimated [19^▪^].

Pocc reflects the airway pressure drop during a full-breath occluded inspiration (i.e., end-expiratory hold maneuver) and thus better reflects inspiratory effort than drive [20]. In a small cohort of patients on assisted ventilation, Pocc was validated as a measure to screen for high Pmus and excessive lung stress [i.e., dynamic transpulmonary driving pressures (ΔP_L_)] using the following formulas [20] and with Pocc measured as negative value:

Predicted Pmus = –3/4 Pocc
Predicted ΔP_L_ = (Ppeak – PEEP) – 2/3 Pocc

Although P0.1 [17] and Pocc [20] were validated against Pes, reflecting global inspiratory muscle effort, recent work [21^▪▪^] focused on correlations of P0.1 and Pocc with specifically diaphragm effort (Pdi) and lung stress and compared their predictive value in the same cohort. P0.1 and Pocc could identify high ΔP_L_ (i.e., >20 cmH_2_O) and the extremes of both low and high Pdi (i.e., Pdi <3 cmH_2_O and >12 cmH_2_O); Pocc outperformed P0.1 in detecting high effort. Similar formulas were found to predict effort and lung stress based on Pocc [20]; hence, if predicted ΔP_L_ is >20 cmH_2_O, lung stress is likely high [5,21^▪▪^].

Neither P0.1 nor Pocc should be used to predict *exact* values for effort or lung distending pressures, considering large between-patient variability in their relationships [17,20,21^▪▪^]. Instead, they are appropriate screening tools. It was recently suggested that both P0.1 and Pocc could predict relapse of respiratory failure in critically ill patients with COVID-19 with high effort [22].

#### Pressure muscle index

The pressure muscle index (PMI), already introduced in 1997 [23], can be calculated during assisted ventilation by performing an end-inspiratory hold and measuring the difference between plateau pressure (Pplat) and peak pressure (Ppeak) (Fig. 2b and c). The inspiratory hold could then unveil the pressure generated by the inspiratory muscles in addition to the ventilator assist: Pplat > Ppeak. Previous small studies showed moderate-to-strong correlations of PMI with Pes-time-product [12,23,24]. Modulating ventilator assist according to PMI targets seems feasible [24,25^▪^]. Docci *et al.*[25^▪^] demonstrated that the individual's response to changing ventilator assist can be divided into two categories: for actively breathing patients, decreasing assist would result in an increased PMI as patients generate more effort in response, whereas in “quasi-passive” patients PMI hardly increases in response to lowering ventilator assist [25^▪^]. This could either be a sign of inspiratory muscle weakness or ventilator over-assistance; in particular, a PMI <0 cmH_2_O (i.e., Pplat < Ppeak) suggests passive insufflation.

Several limitations of PMI monitoring exist. First, its accuracy depends on the readability of the inspiratory hold. Criteria for a reliable Pplat were developed [12]: short time to reach the plateau (<800 ms), duration of a stable plateau for >2 s, and low variation of the plateau (<0.6 cmH_2_O/s) [12]. Especially in patients with high drive and effort, a stable plateau can be hard or even impossible to measure [12,26]; this could itself indicate high effort. Expiratory muscle activity is often present even when there is a clear stable plateau during the inspiratory hold [26]. In this case, the measured airway pressure does not equal to the end-inspiratory elastic recoil pressure. Last, there is no cut-off value for PMI (except that a negative value suggests passive insufflation).

#### Surface electromyography

Being a close surrogate of respiratory drive, the diaphragm's electrical activity, that is, diaphragm electromyography (EMGdi), has often been proposed as a monitoring tool for inspiratory effort. The reference standard is obtained via a dedicated electrodes-embedded nasogastric catheter (also known as EAdi catheter) and a noninvasive alternative is via surface electromyography (sEMGdi) [27^▪^]. EMGdi amplitudes correlate with breathing effort [28–30] but substantial variation between subjects and/or different recordings within a subject (especially in case of sEMGdi [27^▪^,^30^]) makes it unreliable to estimate effort from EMGdi amplitudes only. Hence, a conversion factor is needed: that is, Pmus = *k* × EMGdi. Here, *k* reflects the neuromechanical efficiency and can be obtained from patient-specific measurements, for instance during an end-expiratory occlusion (i.e., *k* = Pocc/EMGdi amplitude during the occlusion) [28,29]. Recent works by Petersen [31] and Grasshoff *et al.*[32^▪^] suggest that mathematical models utilizing the equation of motion could provide an accurate Pmus estimation based on sEMGdi during tidal breathing. Warnaar *et al.*[33] proposed quality criteria for sEMGdi waveforms for reliable effort computation and also found that the conversion factor (Pocc/sEMGdi) depended upon the applied PEEP level [33].

#### Machine learning applications

Mechanical ventilators continuously produce a vast amount of data, yet effort monitoring requires additional diagnostics. Considering technological advances, it is expected that automated algorithms could provide new opportunities by facilitating continuous monitoring and/or identifying those patients who might benefit from additional diagnostics. Few small studies evaluated the flow-time and pressure-time curves. Albani *et al.*[34] proposed the flow index (FI) computed from the concavity of the descending part of the flow waveform: a FI equal to 1 means that the inspiratory flow decreases linearly, a FI <1 represents an upward facing concavity, and a FI >1 a downward facing concavity. The authors found that a FI >1 was associated with increasing effort, while a FI <1 suggests a passive patient. Telias *et al.*[35] developed an algorithm for Pmus computation; this facilitated automated effort detection during synchronous and asynchronous breaths but still requires Pes. De Haro *et al.*[36] analyzed waveforms during square-flow assisted ventilation, where Paw deformation (i.e., flow starvation) is frequently associated with increased effort and/or insufficient flow setting. Using neural network models and Pes as a reference, breaths with strong inspiratory effort were identified [36]. Last, Soundoulounaki *et al.*[37] detected weak efforts by analyzing the flow-time curve during assisted mode. These pilot works illustrate potential but need further validation studies and reference values for predicting low or high effort should be established.

## MONITORING EXPIRATORY MUSCLES

### Relevance

Expiratory muscles are often neglected but frequently engaged in mechanically ventilated patients [10]: active expiration potentially occurs in over two-thirds of adults under assisted ventilation [11^▪▪^,^26^]. Expiratory muscle recruitment enhances expiratory flow and lung emptying, positions the diaphragm more cranially, which benefits tension generation for the next inspiratory effort, and reduces end-expiratory lung volume, thereby creating inspiratory flow upon relaxation. Expiratory muscle contraction can also complicate the management of mechanically ventilated patients. Lung volume reduction may lead to alveolar collapse, cyclical derecruitment, V/Q mismatch, lung elastance and driving pressure deterioration, potentially aggravating lung injury. Indeed, a possible mechanism of oxygenation improvement following neuromuscular blockade in ARDS patients could be the abolishment of expiratory muscle activity [38^▪▪^,^39^]. Expiratory muscle recruitment may also cause dynamic airway collapse [40] – another mechanism of lung inhomogeneity and gas exchange impairment.

### Assessing expiratory muscle effort

**FIGURE 3:**
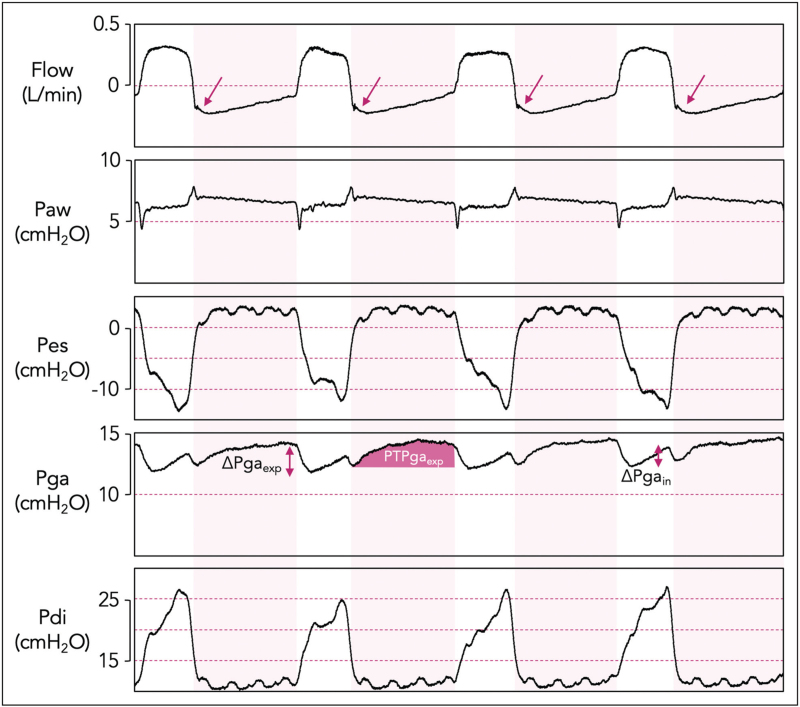
Flow, airway pressure (Paw), esophageal pressure (Pes), gastric pressure (Pga) and the resulting transdiaphragmatic pressure (Pdi = Pga – Pes) in a patient on assisted ventilation and with active use of expiratory muscles. Shaded areas represent the ventilator expiratory phase. Active expiratory muscle use was suspected based on the expiratory flow-time curve (see arrows in flow tracing): the expiratory flow does not decline exponentially but instead increases, creating an upward concavity and flow occasionally fails to return to zero at end-expiration. Expiratory muscle effort was further quantified with Pga monitoring. During inspiration, Pga increases (ΔPga_in_) resulting from diaphragm contraction and the subsequent increase in abdominal pressure. During active expiration, the contraction of the abdominal muscles create an increase in abdominal pressure and thus Pga, demonstrated as ΔPga_exp_. The energy consumption of this expiratory effort can be estimated by the pressure-time product of the expiratory Pga (PTPga_exp_), that is, the area under the expiratory Pga curve.

The first step to identify expiratory muscle recruitment is clinical examination (inspection and palpation of abdominal wall muscles) and observation of expiratory flow-time waveforms (Fig. 3). If, following an initial peak, the expiratory flow does not decline exponentially but instead increases, active expiration should be suspected [11^▪▪^,^41^]. A typical exponential decline in expiratory flow does not rule out the possibility of active expiration [11^▪▪^]. Ultrasound can be used for measuring abdominal wall muscle thickness/thickening [42,43]. In healthy individuals, an increase in abdominal muscle thickness has shown a strong correlation with the muscle's electrical activity [44] and pressure generation [45,46]; the latter was recently confirmed in ICU patients with moderately acceptable reproducibility [45].

An in-depth evaluation is needed for quantification of expiratory effort. In contrast to diaphragm sEMG, recording expiratory muscle sEMG is not widely accessible mostly due to technical challenges, especially in the critically ill [27^▪^]. Gastric pressure (Pga) monitoring is currently the simplest way to measure the force output of the expiratory muscles [13^▪▪^]. When expiratory muscles are active, Pga (and thus pleural pressure) increases during expiration, while upon their relaxation at the beginning of inspiration, Pga rapidly declines. Both expiratory Pga increase (ΔPga_exp_) and Pga decrease at the start of inspiration have been used to detect active expiration [47]. Yet, at early inspiration, Pga may fall either due to expiratory muscle relaxation or contraction of inspiratory muscles. Therefore, ΔPga_exp_ demonstrated better correlation with transversus abdominis EMG than did the early inspiratory Pga decrease [48]. The magnitude of ΔPga_exp_ provides quantification of expiratory muscle effort, while the energy consumption of this effort can be estimated by the pressure-time product of the expiratory Pga (PTPga_exp_). The latter can be derived by calculating the area under the expiratory Pga waveform, with the baseline set as the resting end-expiratory Pga [47] (Fig. 3).

Currently, there are no data to stratify expiratory muscle effort as strong or clinically meaningful based on Pga, but we reason that ΔPga_exp_ ≥1–2 cmH_2_O can be considered active expiration. Notably, there is no method to calibrate gastric balloon filling volume, or measurement accuracy, in contrast to the calibration of Pes [13^▪▪^]. The most accurate way to verify correct catheter position in case of doubt is X-ray.

Eventually, when Pga monitoring is unavailable, intra-abdominal pressure measurement via the urinary catheter is a simple way to identify and roughly quantify expiratory muscle effort. Noninvasive methods to predict the magnitude of expiratory muscle effort have yet to be developed. However, as discussed above, clinical examination, the inspection of expiratory flow-time waveforms and abdominal muscle ultrasound are valuable screening tools to identify the presence of active expiration.

### Impact on inspiratory effort monitoring

Expiratory muscle effort interferes with the interpretation of inspiratory effort and lung stress estimation. Relaxation of the expiratory muscles at the next inspiration decreases pleural pressure; this initial pressure drop should be corrected when calculating inspiratory effort but requires Pga monitoring. Second, Pplat calculation prerequisites passive conditions during end-inspiratory occlusions. Even an apparently flat plateau pressure does not exclude expiratory muscle recruitment, resulting in lung stress overestimation [26]. Moreover, active expiration decreases end-expiratory lung volume below the level corresponding to PEEP. In this setting, airway driving pressure (Pplat – PEEP), underestimates lung stress because part of the measured tidal volume enters the lungs well before the starting point of measurement (PEEP), due to expiratory muscle relaxation. Indeed, a considerable number of patients had ΔP_L_ exceeding airway driving pressure due to this mechanism [49^▪^]. In addition, patient-ventilator asynchronies, traditionally considered as asynchrony between inspiratory effort and ventilator assistance, may be caused by abdominal muscle contraction [11^▪▪^,^50^]. The impact of expiratory muscle recruitment on the reliability of P0.1 and Pocc needs to be studied. Regarding weaning, expiratory muscle activation may indicate weaning failure [47,51].

## CONCLUSION

Different tools are available for monitoring the respiratory muscles’ effort during mechanical ventilation – from noninvasive screening tools to more invasive ways of quantification. This could facilitate individualizing mechanical ventilation within a lung and respiratory muscle-protective approach. Assessment of respiratory muscle effort without taking into account the expiratory muscles is incomplete.

## Acknowledgements

*None.*

*Disclosures: No funding was received for this work. Outside of this work, AJ has received research funding (all to the lab) from ZonMw, Pulmotech B.V., Liberate Medical, Netherlands eScience center and Health∼Holland. Outside of this work, EA has received honoraria for educational lectures and hands-on session from Medtronic.*

### Financial support and sponsorship

*None.*

### Conflicts of interest

*There are no conflicts of interest.*

## REFERENCES AND RECOMMENDED READING

Papers of particular interest, published within the annual period of review, have been highlighted as:

▪ of special interest
▪▪ of outstanding interest
